# Electron Cloaking in MoS_2_ for High-Performance Optoelectronics

**DOI:** 10.1021/acs.nanolett.5c02169

**Published:** 2025-05-28

**Authors:** Yu-Xiang Chen, Jian-Jhang Lee, Ding-Rui Chen, You-Chen Lin, Hao-Ting Chin, Xiu-Yu Huang, Sheng-Kuei Chiu, Chu-Chi Ting, Mario Hofmann, Ya-Ping Hsieh

**Affiliations:** † 71556 Institute of Atomic and Molecular Sciences, Academia Sinica, Taipei, 10617, Taiwan; ‡ International Graduate Program of Molecular Science and Technology, 33561 National Taiwan University, Taipei, 10617, Taiwan; § Molecular Science and Technology Program, Taiwan International Graduate Program, Academia Sinica, Taipei, 10617, Taiwan; ∥ Department of Electronic Engineering, Chung Yuan Christian University, Taoyuan, 320, Taiwan; ⊥ Department of Physics, 597521 National Taiwan University, Taipei, 10617, Taiwan; # Nano Science and Technology Program, Taiwan International Graduate Program, Academia Sinica, Taipei, 10617, Taiwan; 7 Department of Materials Science and Engineering, 34902 Feng Chia University, Taichung, 407, Taiwan; 8 Department of Materials Science, 63460 National University of Tainan, Tainan, 70005, Taiwan; 9 Graduate Institute of Opto-Mechatronics, Department of Mechanical Engineering, National Chung Cheng University, Chia-Yi, 62102, Taiwan

**Keywords:** 2D material, defect-limited mobility, electron cloaking, Coulomb scattering, electron−defect interaction, MoS_2_, vacancies

## Abstract

Defects in two-dimensional (2D) materials represent both challenges and opportunities to their optoelectronic performance. While defects limit the carrier mobility in transistors through increased charge scattering, they also enhance 2D material functionality in sensors. Electron cloaking, a process that reduces Coulomb scattering via localized electron–defect interactions, has recently been shown to mitigate the performance degradation of bulk semiconductors in the presence of defects. We demonstrate the realization of electron cloaking in 2D materials through the metal decoration of defects. Sulfur vacancies were introduced in MoS_2_ and selectively decorated with aluminum using atomic layer deposition. Theoretical and experimental characterization demonstrate the suppression of electronic scattering through localized interactions. Optoelectronic measurements reveal a significant improvement in carrier mobility and lifetime, highlighting the effectiveness of the cloaking mechanism. Our findings open a route independently to maximize performance and functionality of optoelectronic devices, which is illustrated by the realization photosensors with unprecedented sensitivity and speed.

## Graphical abstract

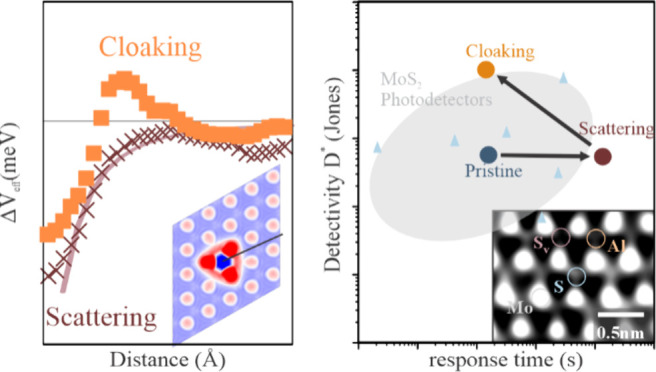

Defects in two-dimensional (2D) materials play a critical role in shaping their electronic and optical properties. The interaction of defects with electrons reduces carrier mobility and carrier lifetime, thus limiting the performance of 2D materials-based electronics and optoelectronic devices.^1^ Consequently, significant effort has been invested in decreasing the defect density and their impact on carrier conduction, such as improved growth techniques^2^ and chemical passivation.^3^ Unfortunately, these approaches fall short in fully eliminating the impact of defects, and 2D materials’ performance remains below expectations.^4^

Paradoxically, modern electronic devices require high levels of defects to achieve the required electrostatic control and conductivity through external doping.^5,6^ Other envisioned 2D materials’ applications are essentially enabled by the presence of defects, including sensing^7–9^ and catalysis,^10^ as they provide tunability of the density of states^11^ and increase the complexity of the 2D material structure.^12^

These developments underscore the need for novel strategies to enhance carrier mobility in the presence of defects. Such an approach would not only mitigate the impact of unavoidable defects in realistic electronic devices but also open up a route to enhancing the performance of sensors and electrocatalysts. A promising avenue to address this issue is based on a recently proposed mechanism, called “electron cloaking”.^13^ Through tailoring of the short-range perturbation produced by suitable dopants, the long-range Coulomb field created by defects can be counteracted, resulting in a decreased overall scattering efficiency (Figure 1(a)).

**Figure 1:**
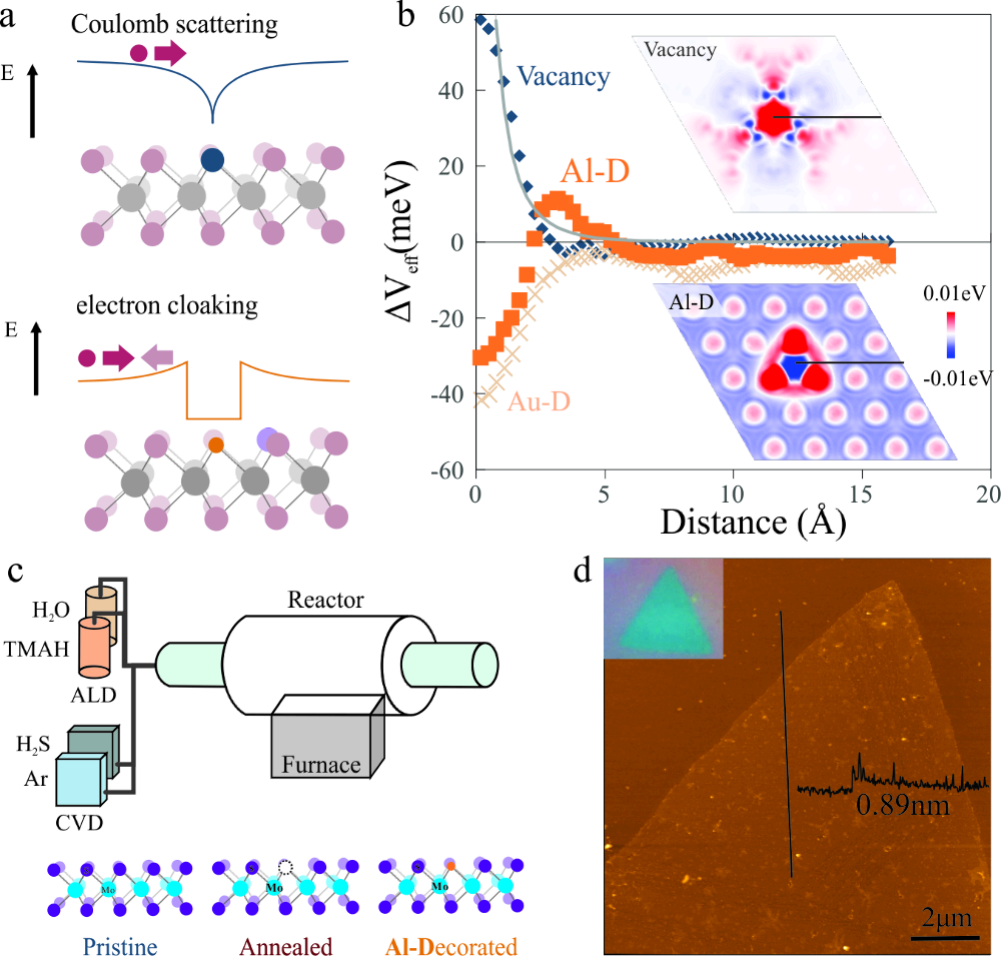
(a) Schematic of Coulombic scattering of charged defects in 2D materials and concept of electron cloaking for metal-decorated vacancies. (b) Simulated effective potential of a vacancy, a Au-decorated and an Al-decorated vacancy in MoS_2_, (insets) spatial distribution of effective potential in the Mo planes for vacancy-defected and Al-decorated MoS_2_ with indication of the cutting line for the plot, (c) setup of combined CVD and ALD precursor systems with indication of different MoS_2_ materials states produced, (d) atomic force micrograph of the MoS_2_ crystal domain with indication of the thickness, indicating its single-layer nature, (inset) corresponding optical micrograph.

In this work, we demonstrate the potential of metal dopants in facilitating electron cloaking of vacancy-defected 2D materials, resulting in enhanced mobility and optoelectronic performance. Molybdenum disulfide (MoS_2_) was controllably supplied with vacancies, due to the prevalence of such defects in realistic materials. Aluminum atoms were shown to selectively decorate these vacancies by atomic layer deposition, as confirmed through comprehensive characterization with atomic-resolution transmission electron microscopy (TEM), X-ray photoelectron spectroscopy (XPS), and electrochemical measurements. Electron cloaking of these Al-decorated vacancies was evidenced by optical spectroscopy, which demonstrated the retention of defect states but a decrease in scattering rates. Carrier transport highlighted the significant enhancement of the mobility through electron cloaking. Our results open up new routes to break the trade-off between performance and functionality of 2D electronic devices, and we highlight this advance through the realization of photosensors with superior abilities.

Sulfur vacancies are one of the most common defects in 2D MoS_2_, due to their low formation energy.^3^ Such defects produce a long-range repulsive Coulomb potential that induces the scattering of conduction electrons.^14,15^ Theoretical work has indicated that Coulomb scattering could be decreased by a short-range potential which introduces an opposing potential component and thus limits the scattering of electrons with the vacancy.^13^

DFT calculations demonstrate the impact of metal decoration on vacancy-induced scattering (Figure 1(b)). A bare vacancy is shown to modify the effective potential of a MoS_2_ layer in a monotonic way that is expected for a screened Coulomb potential in a material (more details on the calculation and effect of dimensionality on the spatial extent of Coulomb potentials are provided in the Supporting Information.) Decoration of the vacancy with gold is shown to introduce an attractive potential due to the lower electronegativity and fewer valence electrons. The resulting potential distribution adheres to the expected Coulomb potential. Aluminum decoration of the vacancy, however, significantly modifies the vacancy potential. At intermediate separations the competition between the vacancy and Al potential distributions results in a nonmonotonic potential distribution. This effect is not visible for gold and demonstrates the impact of aluminum’s small ionic radius on carrier transport: An electron at large distances would experience an attractive force originating from the Al-decoration but could not reach the charged defect due to the repulsive force from the vacancy. This behavior demonstrates the ability of the electron defect interaction (EDI) to mitigate defect scattering and is in agreement with previous work on electron cloaking.^13^ Moreover, the blocking effect of the EDI, i.e., the ratio of center potential to repulsive potential, is higher in our 2D case than in previous reports that focused on bulk materials, which emphasizes the importance of dimensionality on the performance of electron cloaking.^13^ An additional attractive feature of Al is the ability to deposit it in atomically precise quantities and at large scale using atomic layer deposition (ALD) (details on the deposition process including morphology, cycle dependence, and energetics can be found in the Supporting Information).

To test the predicted electron cloaking in Al-doped MoS_2_ vacancies, we need to produce MoS_2_ with a sufficiently high vacancy concentration that can then be decorated by aluminum deposition. However, the high reactivity of sulfur vacancies usually leads to their saturation with environmental contaminants, presenting challenges to our decoration scheme.^16,17^ To overcome this issue, a setup was devised that allows MoS_2_ synthesis, modification, and Al doping without exposure to the ambient (Figure 1(c)). Using this system, we first synthesized MoS_2_ following previous reports.^18^ Large, single-crystalline regions of single-layer MoS_2_ could be synthesized, as confirmed by optical and atomic force microscopy (Figure 1(d)). Then, without breaking vacuum, the sample was annealed at 400 °C for 30 min under a 100 sccm Ar flow in order to increase the density of vacancies.^14^ Finally, the chamber pressure was lowered to 7.2 Torr and Ar flow was introduced during the atomic-layer deposition process.

The vacancy formation and metal decoration processes were investigated by X-ray photoelectron spectroscopy (XPS). The concentration of sulfur vacancies was extracted from the S 2p core level spectrum following previous reports^19^ (Figure 2(a)). It can be seen that intrinsic MoS_2_ exhibits approximately 2% vacancies in agreement with previous reports for CVD-grown material.^3^ Annealing increases the concentration of vacancies to 10%, confirming that heat treatment under a protective atmosphere is a suitable method to increase the number of vacancies in MoS_2_. ALD deposition retains the high concentration of vacancies (9.6%), indicating that defect healing is not substantial.

**Figure 2:**
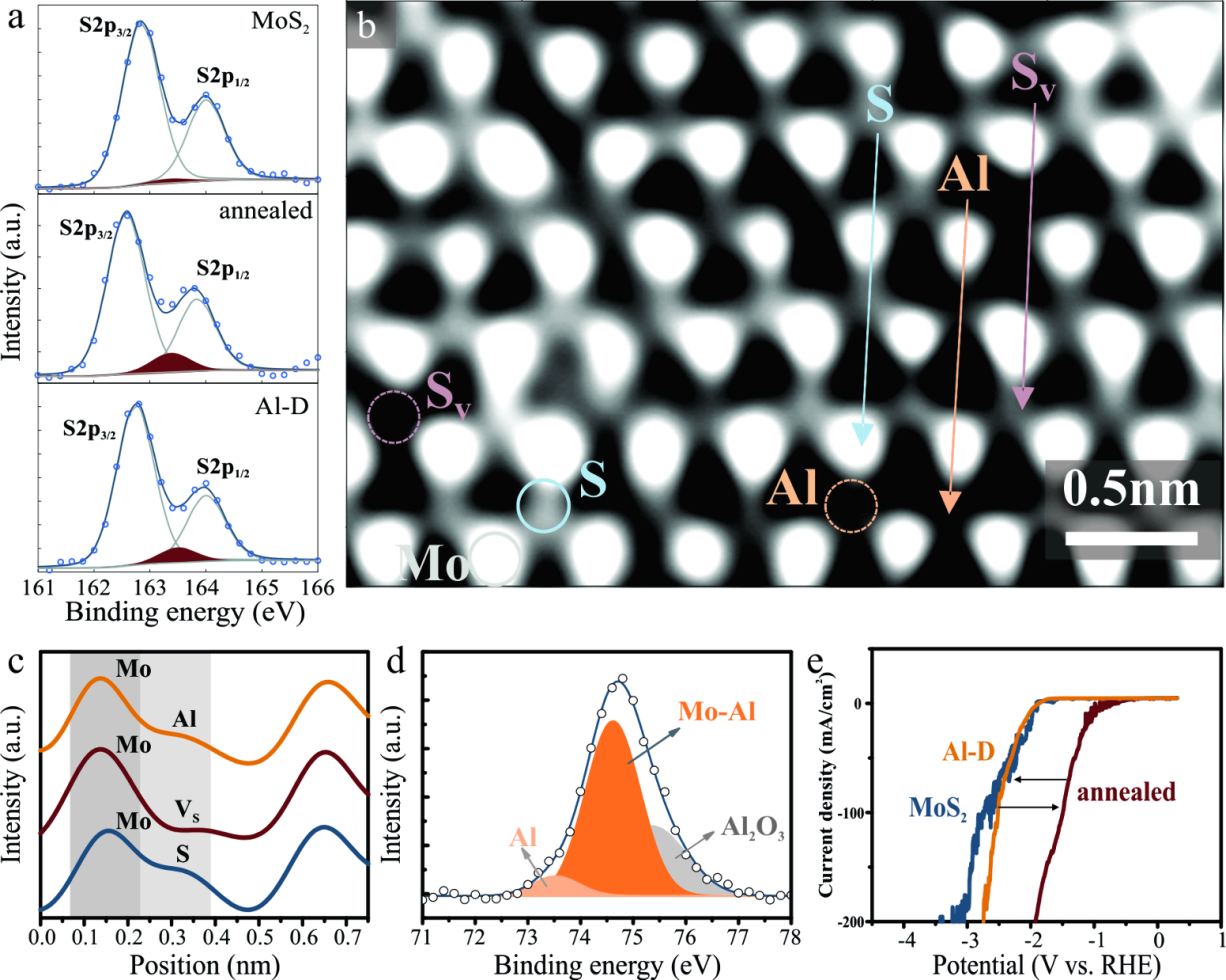
(a) X-ray photoelectron spectrographs of the S 2p peak for different MoS_2_ conditions with indication of the vacancy contribution (the extracted vacancy concentrations are listed in the Supporting Information Table 1); (b) ADF-STEM atomic-resolution image of Al-decorated MoS_2_ with indication of different elements (Mo, Al, S) and sulfur vacancy (S_V_) as extracted from the z-contrast intensity profile; (c) corresponding intensity profile along the lines indicated in (b); (d) XPS spectrum of Al 2p with indication of Al–Mo alloy formation; (e) HER polarization graphs of different MoS_2_ states showing the return of the overpotential to pristine levels after Al-decoration of vacancy-defected MoS_2_.

Atomic-resolution annular dark-field sphere-aberration-corrected STEM was employed to identify the MoS_2_ morphology after of the aluminum deposition process (Figure 2(b)). Due to the difference in atomic number, molybdenum is more visible than sulfur or aluminum. However, analysis of the z-contrast demonstrates the difference between the components. We observed that vacancies and Al-decoration are selectively introduced in the sulfur sublattice (Figure 2(c)). This result confirms that Al will substitute S vacancies, which agrees with our DFT calculations that show that Al-decorated vacancies exhibit a 0.7 eV lower formation energy than bare vacancies. Finally, the selective decoration picture is supported by XPS analysis that demonstrates the formation of Mo–Al bonds (Figure 2(d)).

To confirm the completeness of the Al substitution process throughout the sample, we utilize electrochemical characterization of the hydrogen evolution process, due to the demonstrated sensitivity of this reaction to the presence of vacancies which exhibit higher catalytic activity than pristine MoS_2._ ^8^ We observe a significant decrease in HER overpotential after annealing, indicating the high concentration of catalytically active defects and corroborating previous reports.^8^ The vacancy decoration of aluminum increases the overpotential again, close to the value of intrinsic MoS_2_ (Figure 2(e)). This behavior confirms that a large portion of vacancies are indeed blocked by aluminum and do not contribute to the electrochemical reaction process.

With the selective Al-decoration process established by different spectroscopic and imaging techniques, we investigated its impact on the properties of MoS_2_ as a first step to evaluate its utility in electron cloaking. Previous work demonstrated the charge transfer and formation of strain in Al-decorated MoS_2_ both theoretically.^20,21^ To quantify these effects in our experiments, Raman spectroscopy was utilized due to its sensitivity to electronic and phononic interactions (Figure 3(a)). Representative spectra show the prominence of the A_1g_ and E_2g_ peaks at 403 cm^–1^ and 384 cm^–1^, respectively. The separation of these features agrees with previous reports on single-layer MoS_2._ ^22^

**Figure 3:**
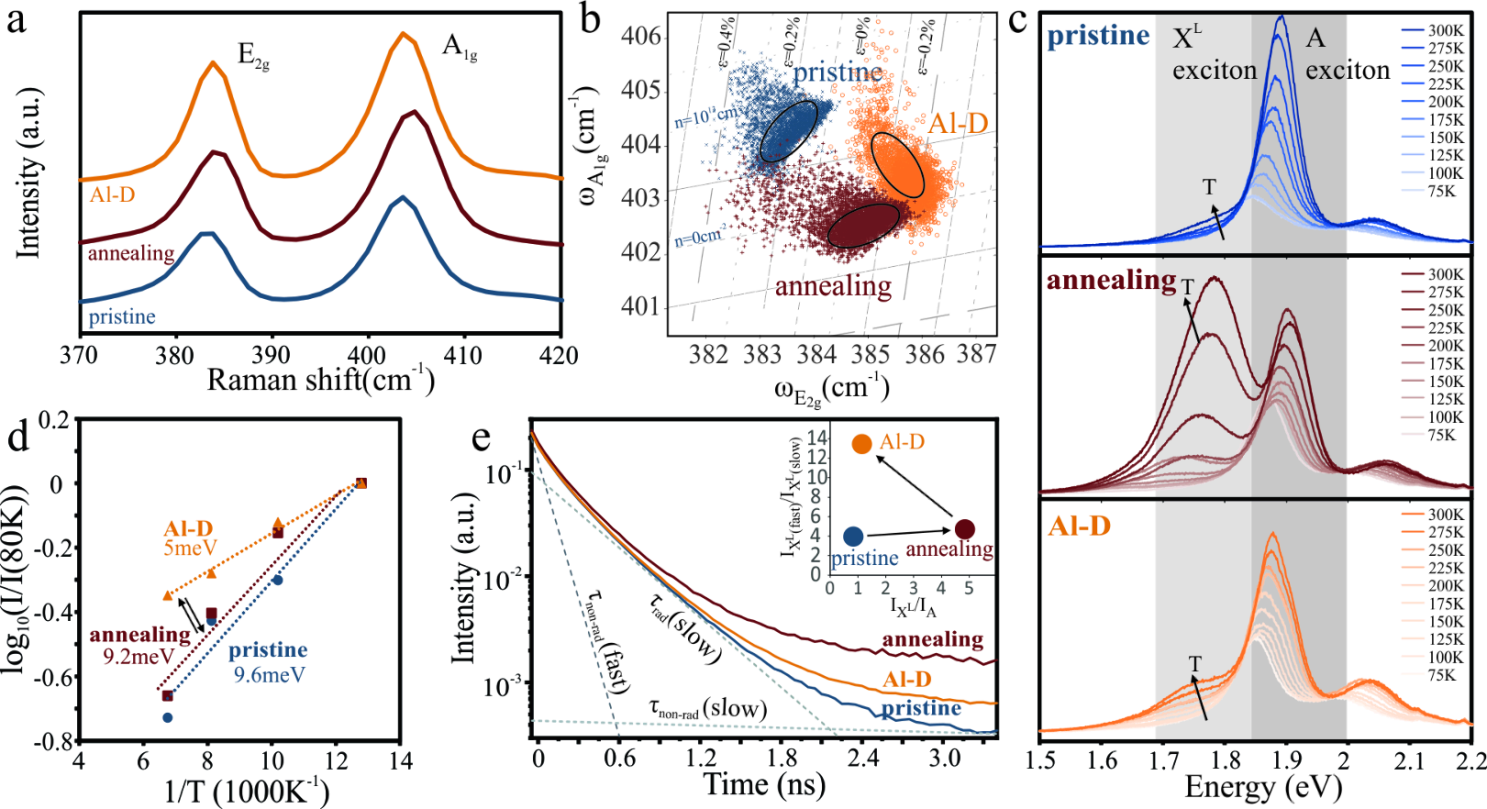
(a) Representative Raman spectra of different MoS_2_ processing conditions; (b) correlation analysis of contributions from strain and doping with the origin being chosen from previous reports;^24^ (c) temperature-dependent photoluminescence spectra for different MoS_2_ conditions with indication of A and X^L^ excitons; (d) Arrhenius plot of X^L^ peak intensity with extracted activation energies; (e) time-resolved photoluminescence spectra with indication of contributions from A and X^L^ recombination pathways, (inset) plot of intensity ratio of radiative X^L^ to A excitons vs intensity ratio of radiative to nonradiative X^L^ exciton.

Correlation analysis of the different shifts of A_1g_ and E_2g_ peak positions with charge transfer and strain can be employed to assess the conditions of MoS_2_ after each step (Figure 3(b)).^23,24^ Upon annealing, we observe a shift in carrier concentration that indicates p-type doping, which agrees with previous reports.^12,13^ Moreover, the vacancy formation process is causing compressive strain relative to the pristine MoS_2_ lattice due to the reorganization of bonds around the defect.^25^ Aluminum decoration is found to reverse some of the charge transfer, which is expected from its electron-donating characteristics. Moreover, the decoration enhances the compressive strain in the lattice, which agrees with the picture of bond reorganization around the vacancy-aluminum structure due to the modified electron defect interaction (EDI). In addition to changing the structural properties and carrier density of MoS_2_, Al-D is expected to modify its electronic properties. Previous reports showed that Al introduces midgap states that are localized around the defect.^20^ Moreover, the introduction of strain is expected to modify the band gap size.^25^ Finally, charge transfer is expected to occur.^26^

To evaluate these effects, we conduct photoluminescence (PL) spectroscopy.^27^ The MoS_2_ PL spectrum shows a prominent peak at 675 nm corresponding to A excitons (Figure 3(c)), which is associated with the direct band gap of monolayer MoS_2._ ^28^ Comparison of the peak positions indicates a red shift of 0.2 eV for annealed MoS_2_ compared to pristine MoS_2_, which confirms the band gap renormalization due to strain. Upon Al-decoration, the original peak position is recovered, suggesting the return to an unstrained condition. This discrepancy between Raman and PL-derived strain values originates from the difference in length scales of phononic and electronic interactions and indicates the strong localization of strain around the defects.^29^

At lower temperatures, a smaller peak at 700 nm becomes noticeable that corresponds to bound excitons (X^L^ or L-band) associated with the states induced by sulfur vacancies. Analysis of the temperature dependence of the X^L^ peak intensity allows us to analyze the energetics of the electron–electron scattering process in the vacancies.^28^ From an Arrhenius plot we extract an activation energy of 9 meV for pristine MoS_2_, which agrees with previous reports^28^ (Figure 3(d)). Annealed samples show an activation energy similar to that of pristine MoS_2_. This observation suggests that the photoluminescence intensity in both cases is limited by thermally activated nonradiative recombination through sulfur vacancies. The increase in vacancy concentration does not change this effect because the dominant channel remains the same.^30^ Based on this analysis, the limited impact of other effects, such as doping and strain, is confirmed.

A significant decrease in activation energy is observed for aluminum-decorated MoS_2_ (Figure 3(d)), in agreement with previous observations on hybridized MoS_2_ defects.^30^ This change in activation energy indicates a decrease in the energy barrier that has to be overcome to initiate nonradiative recombination.^31^ Consequently, the result provides initial evidence of the impact of EDI on the electron scattering potential, which is the prerequisite for the electron cloaking process.

To study the impact of EDI on carrier transport, we conduct time-resolved photoluminescence (TRPL) measurements. The reliability of the measurements was confirmed by a uniform TRPL signal across the MoS_2_ (Supplementary Figure S7). We find that the PL decay curves exhibit contributions from 3 decay processes (Figure 3(e)), which represent the fast and slow decay through the X^L^ exciton and the slow decay of the A exciton.^28^

We quantify their relative contribution by fitting the decay curves to a triple exponential:

$$I\left(t\right)=A_{A_{fast}}e^{-t/{\tau_{A}}_{fast}}+A_{X_{fast}^{L}}e^{-t/\tau_{X_{fast}^{L}}}+A_{X_{slow}^{L}}e^{-t/\tau_{X_{slow}^{L}}}$$ 1

where *A* represents the contribution and τ the lifetime of each excitonic process (details of the fitting process and fitting results are presented in the Supporting Information).

The fast components for the A and X^L^ excitons correspond to the radiative recombination, whereas the slow component represents the phonon-assisted, nonradiative recombination through defects. We employ a correlation analysis of these contributions to distinguish EDI from alternative explanations. Dielectric screening, as observed in thick Al_2_O_3_ films,^32^ would introduce an inverse proportionality between recombination rate and exciton ratio.^33^ Furthermore, changes to the band structure would provide a direct proportionality between exciton concentration and its recombination dynamics.^34^ Finally, an increase in defect density would affect the exciton ratio but not the recombination pathways.

Upon annealing, we observe a behavior that mirrors our temperature-dependent PL analysis: The increased amount of vacancies in annealed MoS_2_ results in a larger concentration of X^L^ excitons. Furthermore, the ratio of radiative to nonradiative recombination of the X^L^ exciton remains similar before and after annealing, which corroborates the similarity in recombination energetics between pristine and annealed MoS_2_ (inset Figure 3(e)). The unique effect of the Al-induced EDI is seen when comparing these results to those of Al-decorated MoS_2_. We observe a significant increase in radiative contribution of the X^L^ exciton at X^L^ to A exciton ratios comparable to those of pristine MoS_2_. This variation in dynamics at similar energetics provides additional evidence that a novel scattering process is being observed.

Carrier transport measurements represent the most direct approach to investigate electron cloaking. We produce back-gated field effect transistors by photolithography (inset of Figure 4(a)). The transconductance curves (Figure 4(a)) show significant variations in threshold voltage and slope between the different MoS_2_ conditions. Pristine MoS_2_ shows an n-type conductance with a strongly negative threshold voltage. Annealing renders the transistor more p-type, as seen by the increase in threshold voltage as expected for vacancy formation and in agreement with our optical characterization. The saturation of vacancies by Al-decoration is confirmed by the change in threshold voltage toward more negative values, resulting in a return to n-type behavior. We utilized Kelvin probe measurements to further quantify the charge transfer process (Figure 4(b)). For pristine MoS_2_ the work function is 4.31 eV, which increases to 4.85 eV for annealing, bringing it close to the intrinsic doping condition (4.86 eV). Al-decoration decreases the work function to 4.42 eV again. These results agree well with our Raman correlation analysis and confirm the efficient saturation of the vacancies by Al-D.

**Figure 4:**
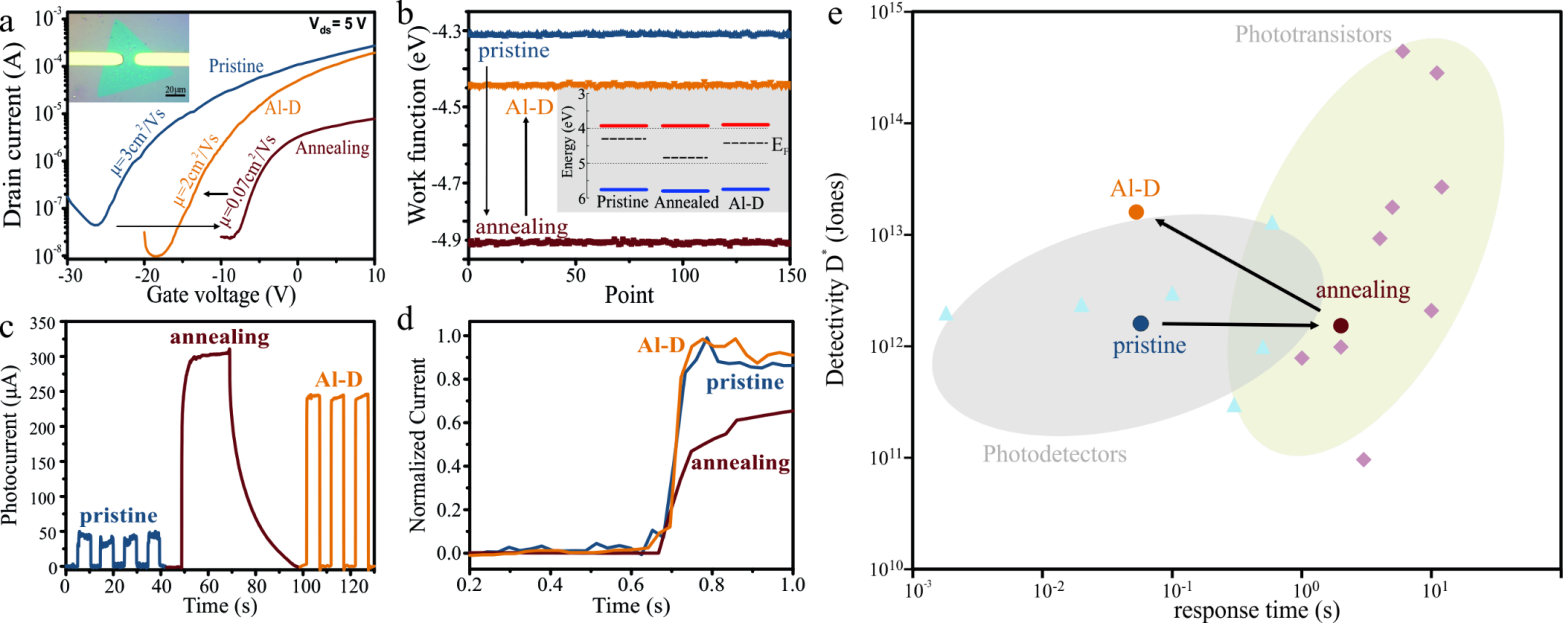
(a) Transconductance curves for FETs for different MoS_2_ states; (inset) representative optical micrograph of an MoS_2_ FET; (b) KP measurements of different MoS_2_ conditions; (inset) schematic of band structure with Fermi level, extracted from data derived from KP, photoemission, and PL; (c) photocurrent at 10 V under toggled 532 nm illumination for different MoS_2_; (d) close-up of on-switching flank of the photocurrent showing recovery of switching time after aluminum decoration; and (e) comparison of specific detectivity and response time of different MoS_2_ photodetectors and phototransistors (more details in the Supporting Information).

The transconductance plots furthermore allow us to extract the field effect mobility, and we find that pristine MoS_2_ has a mobility of 3 cm^2^/(V s). The introduction of vacancies during annealing decreases the mobility by 40 times, emphasizing the high scattering rate of these defects. Aluminum decoration increases the mobility to near-pristine levels.

Combining this trend with our previous characterization results provides clear evidence of the effect of electron cloaking: Our spectroscopic characterization has established that Al-decoration will not significantly decrease the number of vacancies or add large amounts of strain and cannot account for the observed mobility increase. The increased carrier concentration upon Al-D is expected to decrease the mobility. The enhancement of carrier transport in the presence of high defect levels, instead, demonstrates that modification of the EDI can significantly decrease the scattering rate of localized defects. The observed mobility enhancement by an order of magnitude aligns well with the decrease in the electron-vacancy scattering rate extracted by spectroscopy.

The observed high performance at high defect levels emphasizes the potential of electron cloaking. Conventional optimization of electronic devices represents a compromise between increasing carrier density at the cost of increased scattering and reduced mobility. The ability to independently vary these two parameters offers a paradigm shift toward the integration of 2D materials as channels or functional elements in future electronics.^35^ We demonstrate this new ability through photosensors, which usually have to strike a balance between sensitivity and device performance.^5^

We utilize photolithography to produce homogeneous MoS_2_ photodetectors on SiO_2_ and illuminate them at 532 nm. The time-dependent photocurrent under pulsed illumination shows significant differences under the different conditions (Figure 4(c)). Pristine MoS_2_ shows a small photocurrent and fast response time, as expected for MoS_2_ due to its high carrier mobility and low external quantum efficiency.^36^ The response of annealed MoS_2_ demonstrates the impact of vacancies on the sensor’s response. The increased photocurrent indicates the higher quantum yield, while the long decay time corroborates the decreased mobility due to increased Coulombic scattering. The Al-decorated device emphasizes the impact of electron cloaking by combining the high sensitivity imparted by vacancies with a short response time brought about by decreased scattering. Closer inspection reveals that the increased device conductivity, i.e., the product of mobility and carrier concentration, decreases the RC time constant of Al-decorated photodetectors below the pristine case (Figure 4(d)).

To quantify this observation, we evaluated the specific detectivity of each photosensor. This figure of merit represents a combination of sensitivity and operation bandwidth. Indeed, we see that previous work shows a clear trend between detectivity and response time (Figure 4(e)). Our pristine device exhibits a detectivity that is comparable to previous MoS_2_ devices illuminated under visible light. The aforementioned trade-off between speed and sensitivity leads to similar performance of the annealed device. However, the impact of electron cloaking in reducing the response time while retaining the sensitivity leads to our Al-decorated MoS_2_ photosensor exhibiting one of the highest reported detectivities of MoS_2_ to date (a comparison to literature is provided in the Supporting Information).

Finally, the detectivity can be employed as a predictor of the effectiveness of electron cloaking in Al-decorated MoS_2_ with different Al thicknesses. As expected from the EDI picture, we find that a decrease in Al-D coverage enhances the electron cloaking, as vacancy saturation only requires a single atomic layer and spurious aluminum will act as additional Coulombic scatterers (more information can be found in the Supporting Information).

We have established the impact of electron cloaking on enhancing the performance of vacancy-defected MoS_2_. Upon annealing, a large density of vacancies can be introduced in MoS_2_, which controls the optical and carrier transport properties of MoS_2_ through Coulomb scattering, as established by comprehensive spectroscopic and microscopic characterization. Aluminum decorates the vacancies, modifying the electron–defect interaction and decreasing the Coulomb potential. A significant increase in mobility demonstrates the particular potential of electron cloaking for 2D materials as a viable pathway for overcoming defect-limited mobility. Our approach is expected to be applicable to a wide range of 2D materials and dopants and opens new avenues for high-performance optoelectronic devices.

## Supplementary Material



## Notes

Additional experimental details, materials, and methods, including atomic force and scanning electron micrographs; extended characterization, including Raman and TRPL mapping data, XPS, and valence band photoemission spectra; details on simulation and fitting processes; literature overview of MoS_2_ photodetector performance (PDF)
