# Supplementary material for: Electron Cloaking in MoS2 for High-Performance Optoelectronics
Source: Nano Lett. 2025 May 28;25(23):9463–9. doi: 10.1021/acs.nanolett.5c02169 (PMC12164522; doi:10.1021/acs.nanolett.5c02169)
Supplement: Supplementary file 1 [file nl5c02169_si_001.pdf]

# Supporting Information

# Electron Cloaking in $MoS_2$ for High-Performance Optoelectronics

*Yu-Xiang Chen[1,2,3], Jian-Jhang Lee[1], Ding-Rui Chen[4], You-Chen Lin[5,6], Hao-Ting Chin[1,2,3], Xiu-Yu Huang[7], Sheng-Kuei Chiu[8], Chu-Chi Ting[9], Mario Hofmann[5], and Ya-Ping Hsieh[1*]*

[1]Institute of Atomic and Molecular Sciences, Academia Sinica, Taipei 10617, Taiwan

[2]International Graduate Program of Molecular Science and Technology, National Taiwan University, Taipei 10617, Taiwan

[3]Molecular Science and Technology Program, Taiwan International Graduate Program, Academia Sinica, Taipei 10617, Taiwan

[4] Department of Electronic Engineering, Chung Yuan Christian University, Taoyuan 320, Taiwan

[5]Department of Physics, National Taiwan University, Taipei 10617, Taiwan

[6]Nano Science and Technology Program, Taiwan International Graduate Program, Academia Sinica, Taipei 10617, Taiwan

[7]Department of Materials Science and Engineering, Feng Chia University, Taichung 407, Taiwan

[8]Department of Materials Science, National University of Tainan, Tainan 70005, Taiwan

[9]Graduate Institute of Opto-Mechatronics, Department of Mechanical Engineering, National Chung Cheng University, Chia-Yi 62102, Taiwan

## Growth method

Molybdenum disulfide ($MoS_2$) was synthesized on an $SiO_2$/Si substrate using chemical vapor deposition (CVD). The substrate was first cleaned and treated with oxygen plasma, followed by spin-coating of a sodium chloride (NaCl) solution to assist the growth. $MoO_3$ was deposited onto graphite paper using electron beam evaporation and then stacked face-to-face with the prepared substrate. Hydrogen sulfide ($H_2S$) gas was introduced for the sulfurization reaction, and $MoS_2$ was formed at 900°C under a controlled temperature program.

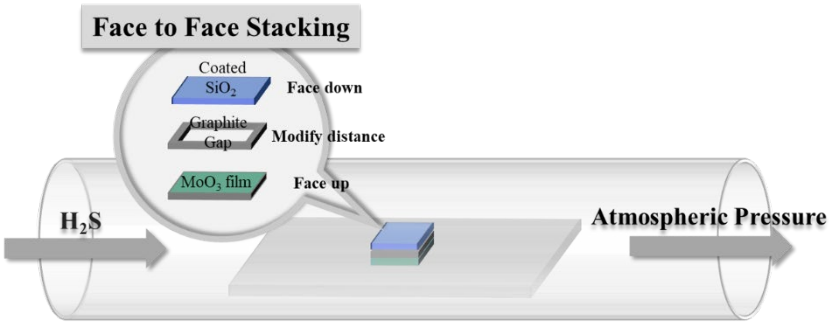


***Figure S1. Schematic diagram of the preparation of molybdenum disulfide by chemical vapor deposition.***

## Annealing to create defect

To create sulfur-rich vacancies, the previously synthesized molybdenum disulfide was thermally annealed at 400°C for 30 minutes under 100sccm Argon flow.

## Aluminum decoration

Aluminum decoration was achieved via atomic layer deposition (ALD) performed after annealing without breaking the vacuum. The process consisted of 50 cycles, with each

cycle involving 0.1s exposure to trimethylaluminum (TMAH), followed by 1s exposure to water vapor, and continuous Ar purging at 200°C. TMAH and water were used as precursors on $MoS_2$ during the 50-cycle process at a switching frequency of 50 cycles[1].

**Photoluminescence characterization**

Temperature-dependent photoluminescence was acquired on a Raman System (NANOBASE, NBOS-220012) in a temperature control stage (Linkam, LNP96-S). The laser power for each PL measurement was set to 10 mW, unless otherwise specified.

**Fermi-level measurement**

The work function of $MoS_2$ was measured using a Kelvin probe technique with the APS01 (KP Technology) system, while the valence band maximum was determined via photoemission. By combining these values with the band gap obtained from light emission, the full band structure was derived.

**Computational method**

The work function of $MoS_2$ was We further investigated the formation energy of S vacancies in $MoS_2$ and Al-decorated $MoS_2$. We used DFTB+[2] with GFN2-xTB

Hamiltonian[3] for the calculations. The effective potential was calculated using the linear combination of atomic orbitals (LCAO) model.

**DFT simulation details and fitting**

First, we constructed pristine $MoS_2$, $MoS_2$ with an S vacancy, and Al-decorated $MoS_2$. We calculated the energy of each system, as well as single S and Al atoms for the formation energy calculations. The formation energy was obtained using the following equations:

$$E_{form} = E_{tot} - \Sigma_x E_{tot}(x)$$

We found that the formation energy of the S-vacancy is about 4.6 eV per vacancy, indicating that energy is required to form the vacancy. On the other hand, the formation energy for Al-decorated $MoS_2$ is about -3.9 eV per vacancy. This suggests that Al atoms tend to fill the vacancy sites, as doing so lowers the system's energy. This result is consistent with our experimental observations which Al will fill the S vacancies.

The effective potential difference was obtained by subtracting the vacancy effective potential from the pristine effective potential. A cutting line was placed by intersecting the Mo-plane and the xz plane at the location of the vacancy.

The potential distribution of the bare vacancy was fitted to theoretical predictions for two different potentials. The Thomas-Fermi potential represents the potential of a screened charge in 3D and follows the equation

$$V_{TF} = \frac{e}{4\pi\varepsilon_r\varepsilon_0 r} e^{-r/r_D}$$

, where $r_D$ is the screening length.

The screened 2D case was described by the full-form Rytova-Keldysh potential.

$$V_{RK} = \frac{e}{r_0}\frac{\pi}{2}\left[H_0\left(\frac{\kappa r}{r_D}\right) - Y_0\left(\frac{\kappa r}{r_D}\right)\right]$$

, where $\kappa$ is the environmental dielectric constant[4].

Fitting to both functional forms shows reasonable agreement with the bare vacancy potential and the extracted parameters are consistent for both cases with screening lengths in the Angstrom range and a relative permittivity in the order of 100.

The influence of different charge states was investigated by DFTB and we find less than 10% variation in the charge distribution between the neutral and singly negatively charged case.

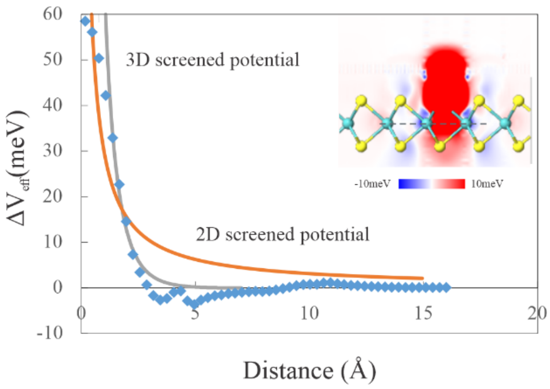


***Figure S2. Calculated effective potential vs distance along a cutting line extending from the vacancy as shown in Figure 1(b)) with fits for 3D screened potential and 2D potential according to, the text (inset) overlay of $V_{eff}$ spatial distribution and $MoS_2$ structure with indication of Mo cutting plane***

***Table 1. Comparison of charge states upon change of overall charge state.***

| | **Neutral** | **Negatively charged** |
|---|---|---|
| **vacancy** | *Mo = –0.86 to –1.11,*<br>*S = 0.56 to 0.58* | *Mo = –0.84 to –1.06,*<br>*S = 0.62 to 0.63* |
| **Al-D** | *Mo = –0.97 to –1.16,*<br>*S = 0.57 to 0.59,*<br>*Au = –1.22* | *Mo = –0.44 to –1.02,*<br>*S = 0.65 to 0.66,*<br>*Al = –1.36* |
| **Au-D** | *Mo = –0.48 to –1.03,*<br>*S = 0.60 to 0.62,*<br>*Al = –1.40* | *Mo = –0.95 to –1.13,*<br>*S = 0.62 to 0.63,*<br>*Au = –1.13* |

**X-ray photoelectron spectroscopy of Mo core-level**

The formation of vacancies and the metal decoration process were studied using X-ray photoelectron spectroscopy (XPS). According to previous reports[5], the concentration of sulfur vacancies was extracted from the Sulfur core-level spectra. An initial vacancy concentration of 4% was derived from the S 2s spectra, which increased to 6% after annealing. Finally, after aluminum decoration, the vacancy concentration returned to 4%. Consistent with previous findings, the metal decoration process effectively mitigates the generation of vacancies.

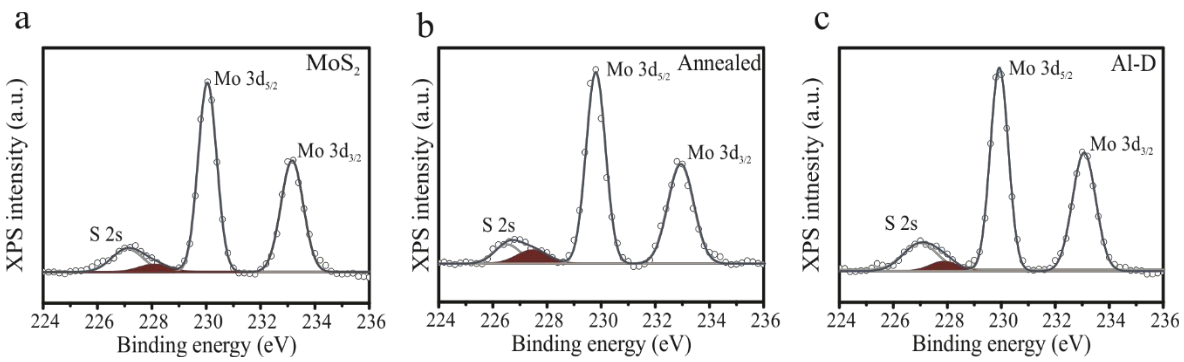


***Figure S3. X-ray photoelectron spectra of Mo 3d peaks: (a)MoS$_2$ (b)Annealed (c) Al-Decorated.***

***Table 2. Summary of the peak area of each peak.***

| | Mo 3d | | | | S 2p | | |
|---|---|---|---|---|---|---|---|
| | S 2s | **defect** | Mo 3d$_{5/2}$ | Mo 3d$_{3/2}$ | S 2P$_{3/2}$ | **defect** | S 2P$_{1/2}$ |
| MoS$_2$ | 542 | **174** | 2698 | 1918 | 1261 | **27** | 639 |
| Annealed | 306 | **243** | 2203 | 1527 | 937 | **100** | 426 |
| Al-D | 651 | **186** | 2679 | 2019 | 1248 | **120** | 528 |

**SEM image after Al-decoration**

A phenomenon also observed in the SEM image. Previous reports have demonstrated that the MoS$_2$ surface is hydrophobic and therefore after Al-D process, alumina is anticipated to be formed at defect sites[1], which are augmented by thermal annealing to allow Al-D precursors to adsorb on sulfur vacancies.

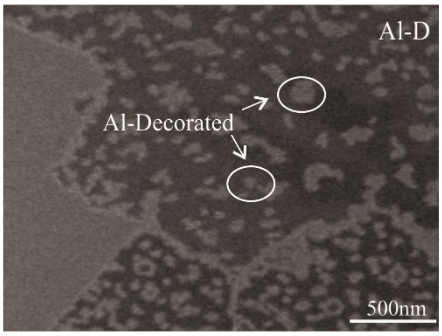


***Figure S4. Scanning electron microscope images of Al-Decorated.***

**AFM image after Al-decoration**

Atomic force microscope scans reveal distinct differences in the surface morphology of pristine $MoS_2$ compared to after Al decoration. Following Al-D process, numerous Al are deposited on the $MoS_2$ surface.

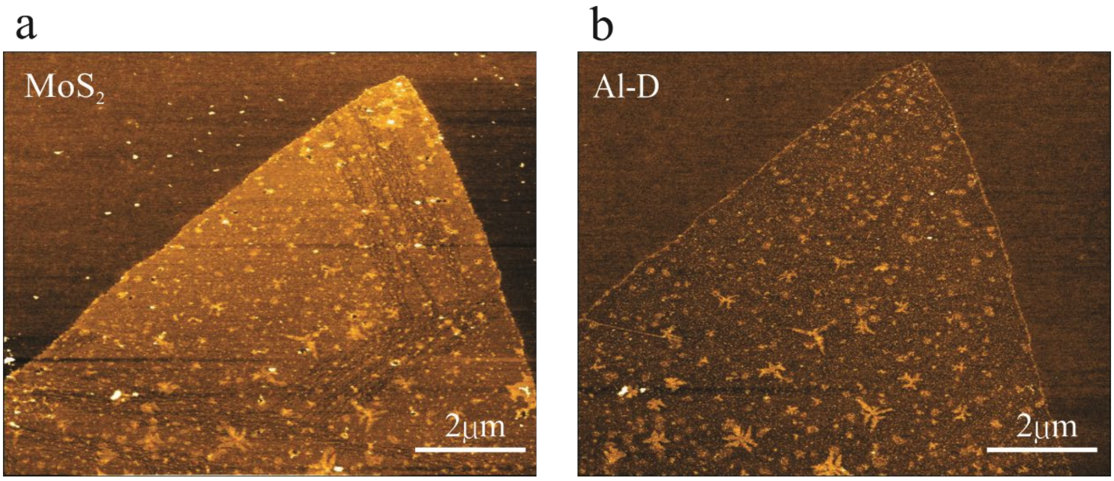


***Figure S5. AFM image of (a)$MoS_2$ (b) Al-Decorated.***

**Time-resolved photoluminescence fitting**

To extract the contributions of $X^L$ and A exciton recombination, we fitted the TRPL spectra using least-square fitting routines. The underestimation of the low-intensity tail was overcome by scaling the y-axis logarithmically first and then fitting

$$\log_{10} I = \log_{10}(A_1 * exp(-t/\tau_1) + A_2 * exp(-t/\tau_2) + A_3 * exp(-t/\tau_3))$$

*Table 3. Summary of extracted fitting parameters.*

| | Value and 95% confidence interval | | |
|---|---|---|---|
| | **Pristine** | **Annealed** | **Al-D** |
| **$A_1$** | $44.65 \pm 3.9$ | $9.1 \pm 4$ | $136.7 \pm 12$ |
| **$A_2$** | $37.3 \pm 2$ | $44.5 \pm 5$ | $156.4 \pm 6$ |
| **$A_3$** | $9.43 \pm 0.9$ | $9.5 \pm 0.2$ | $11.6 \pm 0.03$ |
| $\boldsymbol{\tau_1}$ | $0.1 \pm 0.03ns$ | $0.1 \pm 0.05ns$ | $0.1 \pm 0.04ns$ |
| $\boldsymbol{\tau_2}$ | $0.42 \pm 0.02ns$ | $0.42 \pm 0.04ns$ | $0.34 \pm 0.01ns$ |
| $\boldsymbol{\tau_3}$ | $15 \pm 0.2ns$ | $14.93 \pm 0.4ns$ | $12 \pm 0.3ns$ |

**Air Photoemission System measurement**

Work function and valence band maximum measurements of $MoS_2$ were conducted using an APS01 (KP Technology) system with the Kelvin-probe technique and photoemission, respectively. These values, combined with the band gap obtained from photoemission or light emission, allowed for the derivation of the full band structure. After annealing, the maximum valence band of pristine $MoS_2$ shifts from 5.8 eV to 5.76 eV and returns to 5.8 eV following Al decoration, which is consistent with previous reports.

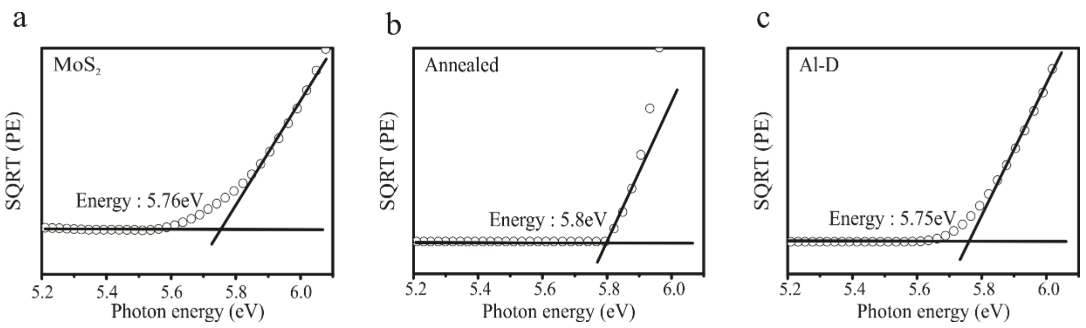


***Figure S6. Photoelectron emission of valence band edge of (a) $MoS_2$ (b) Annealed (c) Al-Decorated.***

*Table 4. Electronic Properties of $MoS_2$, Annealed $MoS_2$, and Al-decorated $MoS_2$.*

| | **Valence band minimum** | **Work function** | **Band Gap** | **Type** |
|---|---|---|---|---|
| **$MoS_2$** | 5.76eV | 4.31eV | 1.83eV | n-type |
| **Annealed** | 5.80eV | 4.85eV | 1.87eV | p-type |
| **Al-D** | 5.75eV | 4.42eV | 1.85eV | n-type |

**Transfer and device fabrication**

$MoS_2$ was coated with PMMA and soaked in 1M NaOH until the PMMA/$MoS_2$ layer floated. The layer was then washed three times with DI water and transferred onto a $Si_3N_4/SiO_2$ substrate. PMMA was removed using acetone, and a nitrogen gun was used to eliminate moisture, completing the transfer. The transferred material was patterned via photolithography. Electrodes were fabricated with 40 nm of gold using thermal evaporation. A silicon wafer served as the gate, and current-voltage characteristics were measured with a Keysight B2912A meter. The field-effect mobility (μ) of the FET was calculated using the equation:

$$\mu = \frac{dI_{ds}}{dV_{gs}} \times \frac{L}{W} \times \frac{1}{C_g V_{ds}}$$

**Evolution of $MoS_2$ Raman Spectrum features**

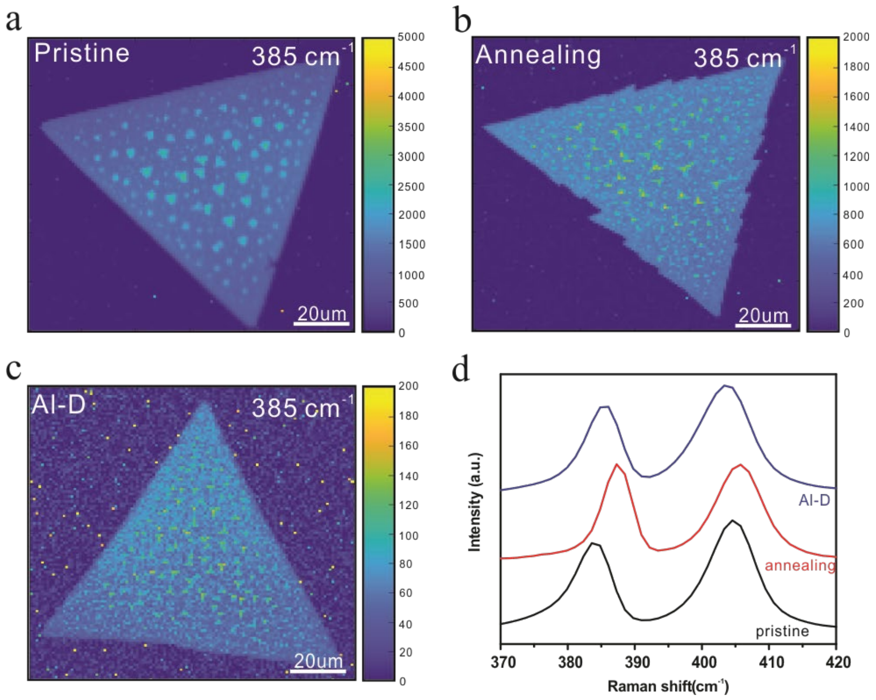


***Figure S7. Raman mapping of (a) pristine, (b) annealed, and (c) Al-D $MoS_2$ samples. (d) Averaged Raman spectra from over 5000 measurements under different conditions.***

**Evolution of difference condition $MoS_2$ TRPL mapping**

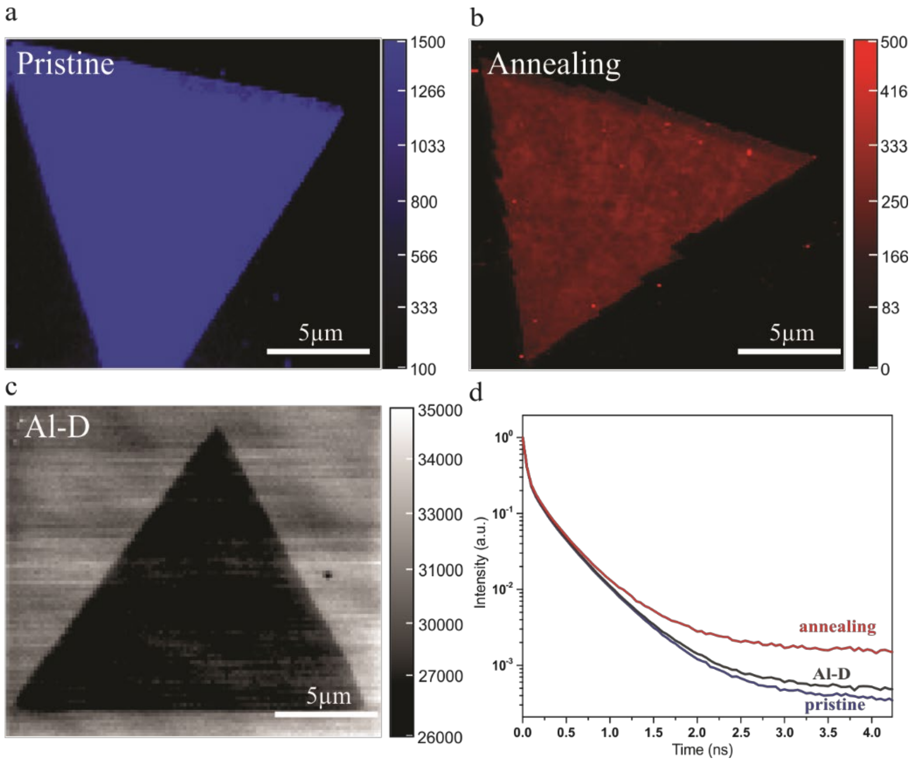


***Figure S8. Time-Resolved Photoluminescence (TRPL) mapping of (a) pristine $MoS_2$, (b) annealed $MoS_2$, (c) Al-D $MoS_2$. (d) TRPL decay curves of pristine, annealed, and Al-D $MoS_2$ averaged over 10,000 spectrums.***

## Surface topography evolution with increasing Al-D Cycles

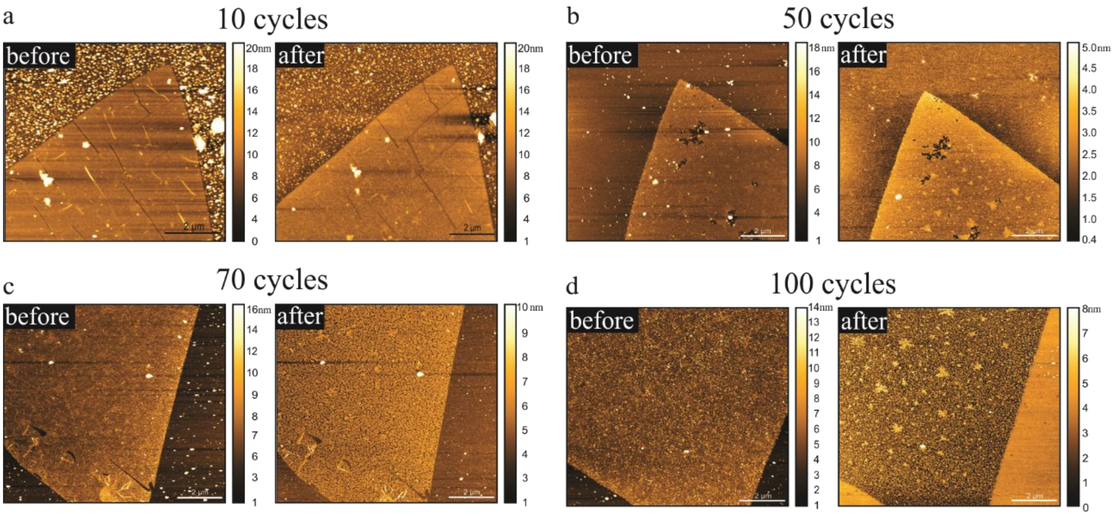


***Figure S9. Atomic Force Microscopy (AFM) images of $MoS_2$ deposited via Al-D with varying cycle numbers. (a) 10 cycles, (b) 50 cycles, (c) 70 cycles, and (d) 100 cycles.***

## Effect of Al-D cycle number on the photocurrent of $MoS_2$

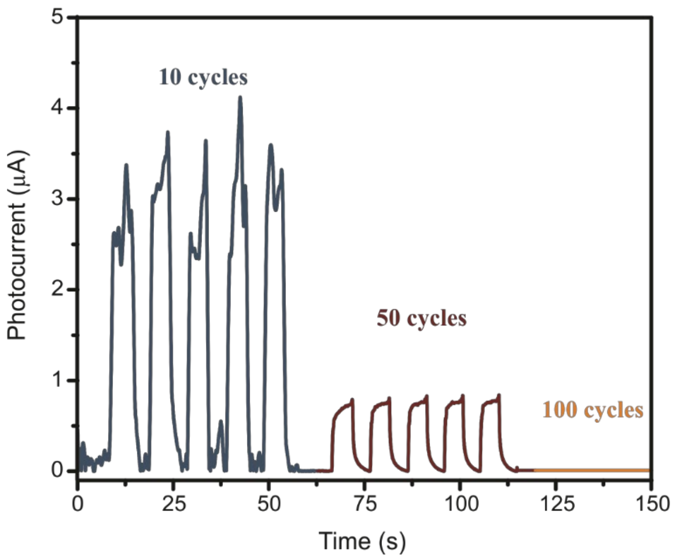


***Figure S10. Photocurrent of $MoS_2$ with different Al-D cycles.***

***Table 5. Comparison of response time and detectivity in $MoS_2$-based photodetectors with different fabrication methods.***

| | Method | Response time(s) | Detectivity | Ref. |
|---|---|---|---|---|
| **$MoS_2$** | Exfoliated | 4 | 2.0E+09 | [6] |
| **$MoS_2$** | Exfoliated | 0.04 | 1.7E+09 | [7] |
| **$MoS_2$** | Sputter | 0.3 | 5.0E+08 | [8] |
| **$MoS_2$** | CVD | 0.23 | 2.6E+13 | [9] |
| **$MoS_2$** | CVD | 31 | 2.3E+12 | [10] |
| **$MoS_2$** | CVD | 0.35 | 7.2E+11 | [11] |
| **$MoS_2$** | CVD | 0.078 | 1.0E+14 | [12] |
| **$MoS_2$** | CVD | 0.74 | 5.7E+12 | [13] |
| **Multi-layer $MoS_2$** | CVD | 0.02 | 4.2E+08 | [14] |
| **Nb/$MoS_2$** | CVD | 4.9 | 5.0E+12 | [15] |
| **ZnPc/$MoS_2$** | CVD | 0.1 | 1.0E+11 | [16] |
| **QDs/$MoS_2$** | Hydrothermal | 0.3 | 2.7E+12 | [17] |
| **SnS/$MoS_2$** | Hydrothermal | 0.12 | 3.2E+11 | [18] |
| **PbS/$MoS_2$** | Hydrothermal | 0.3 | 1.0E+11 | [19] |
| **Al-D $MoS_2$** | CVD | 0.057 | 1.0E+13 | This study |

**Reference**

(1) Kropp, J. A.; Sharma, A.; Zhu, W.; Ataca, C.; Gougousi, T. Surface Defect Engineering of MoS2 for Atomic Layer Deposition of TiO2 Films. *ACS Applied Materials & Interfaces* **2020**, *12* (42), 48150-48160. DOI: 10.1021/acsami.0c13095.

(2) Hourahine, B.; Aradi, B.; Blum, V.; Bonafé, F.; Buccheri, A.; Camacho, C.; Cevallos, C.; Deshaye, M. Y.; Dumitrică, T.; Dominguez, A.; et al. DFTB+, a software package for efficient approximate density functional theory based atomistic simulations. *The Journal of Chemical Physics* **2020**, *152* (12). DOI: 10.1063/1.5143190 (acccessed 5/13/2025).

(3) Bannwarth, C.; Ehlert, S.; Grimme, S. GFN2-xTB—An Accurate and Broadly Parametrized Self-Consistent Tight-Binding Quantum Chemical Method with Multipole Electrostatics and Density-Dependent Dispersion Contributions. *Journal of Chemical Theory and Computation* **2019**, *15* (3), 1652-1671. DOI: 10.1021/acs.jctc.8b01176.

(4) Xiao, K.; Kan, C.-M.; Parkin, S.; Cui, X. Coulomb potential screening via charged carriers and charge-neutral dipoles/excitons in two-dimensional case. *arXiv preprint arXiv:2309.14101* **2023**.

(5) Syari'ati, A.; Kumar, S.; Zahid, A.; Ali El Yumin, A.; Ye, J.; Rudolf, P. Photoemission spectroscopy study of structural defects in molybdenum disulfide (MoS2) grown by chemical vapor deposition (CVD). *Chemical Communications* **2019**, *55* (70), 10384-10387, 10.1039/C9CC01577A. DOI: 10.1039/C9CC01577A.

(6) Lopez-Sanchez, O.; Lembke, D.; Kayci, M.; Radenovic, A.; Kis, A. Ultrasensitive photodetectors based on monolayer MoS2. *Nature Nanotechnology* **2013**, *8* (7), 497-501. DOI: 10.1038/nnano.2013.100.

(7) Tang, X.; Wang, S.; Liang, Y.; Bai, D.; Xu, J.; Wang, Y.; Chen, C.; Liu, X.; Wu, S.; Wen, Y.; et al. High-performance, self-powered flexible MoS2 photodetectors with asymmetric van der Waals gaps. *Physical Chemistry Chemical Physics* **2022**, *24* (12), 7323-7330, 10.1039/D1CP05602F. DOI: 10.1039/D1CP05602F.

(8) Ling, Z. P.; Yang, R.; Chai, J. W.; Wang, S. J.; Leong, W. S.; Tong, Y.; Lei, D.; Zhou, Q.; Gong, X.; Chi, D. Z.; et al. Large-scale two-dimensional MoS2 photodetectors by magnetron sputtering. *Opt. Express* **2015**, *23* (10), 13580-13586. DOI: 10.1364/OE.23.013580.

(9) Vu, Q. A.; Lee, J. H.; Nguyen, V. L.; Shin, Y. S.; Lim, S. C.; Lee, K.; Heo, J.; Park, S.; Kim, K.; Lee, Y. H.; et al. Tuning Carrier Tunneling in van der Waals Heterostructures for Ultrahigh Detectivity. *Nano Lett* **2017**, *17* (1), 453-459. DOI: 10.1021/acs.nanolett.6b04449 From NLM.

(10) Liu, H.; Gao, F.; Hu, Y.; Zhang, J.; Wang, L.; Feng, W.; Hou, J.; Hu, P. Enhanced photoresponse of monolayer MoS2 through hybridization with carbon quantum dots

as efficient photosensitizer. *2D Materials* **2019**, *6* (3), 035025. DOI: 10.1088/2053-1583/ab1c20.
(11) Li, Y.; Li, L.; Li, S.; Sun, J.; Fang, Y.; Deng, T. Highly Sensitive Photodetectors Based on Monolayer MoS2 Field-Effect Transistors. *ACS Omega* **2022**, *7* (16), 13615-13621. DOI: 10.1021/acsomega.1c07117.
(12) Jian, J.; Chang, H.; Dong, P.; Bai, Z.; Zuo, K. A mechanism for the variation in the photoelectric performance of a photodetector based on CVD-grown 2D MoS2. *RSC Advances* **2021**, *11* (9), 5204-5217, 10.1039/D0RA10302K. DOI: 10.1039/D0RA10302K.
(13) Paul, K. K.; Mawlong, L. P. L.; Giri, P. K. Trion-Inhibited Strong Excitonic Emission and Broadband Giant Photoresponsivity from Chemical Vapor-Deposited Monolayer MoS(2) Grown in Situ on TiO(2) Nanostructure. *ACS Appl Mater Interfaces* **2018**, *10* (49), 42812-42825. DOI: 10.1021/acsami.8b14092 From NLM.
(14) Lee, Y.; Yang, J.; Lee, D.; Kim, Y.-H.; Park, J.-H.; Kim, H.; Cho, J. H. Trap-induced photoresponse of solution-synthesized MoS2. *Nanoscale* **2016**, *8* (17), 9193-9200, 10.1039/C6NR00654J. DOI: 10.1039/C6NR00654J.
(15) Kim, Y.; Bark, H.; Kang, B.; Lee, C. Wafer-Scale Substitutional Doping of Monolayer MoS2 Films for High-Performance Optoelectronic Devices. *ACS Applied Materials & Interfaces* **2019**, *11* (13), 12613-12621. DOI: 10.1021/acsami.8b20714.
(16) Huang, Y.; Zhuge, F.; Hou, J.; Lv, L.; Luo, P.; Zhou, N.; Gan, L.; Zhai, T. Van der Waals Coupled Organic Molecules with Monolayer MoS2 for Fast Response Photodetectors with Gate-Tunable Responsivity. *ACS Nano* **2018**, *12* (4), 4062-4073. DOI: 10.1021/acsnano.8b02380.
(17) Mukherjee, S.; Jana, S.; Sinha, T. K.; Das, S.; Ray, S. K. Infrared tunable, two colour-band photodetectors on flexible platforms using 0D/2D PbS–MoS2 hybrids. *Nanoscale Advances* **2019**, *1* (8), 3279-3287, 10.1039/C9NA00302A. DOI: 10.1039/C9NA00302A.
(18) Selamneni, V.; Sukruth, S.; Sahatiya, P. Performance Enhancement of Highly Flexible SnS(p)/MoS2(n) Heterostructure based Broadband Photodetector by Piezo-phototronic Effect. *FlatChem* **2022**, *33*, 100379. DOI: https://doi.org/10.1016/j.flatc.2022.100379.
(19) Kufer, D.; Nikitskiy, I.; Lasanta, T.; Navickaite, G.; Koppens, F. H. L.; Konstantatos, G. Hybrid 2D–0D MoS2–PbS Quantum Dot Photodetectors. *Advanced Materials* **2015**, *27* (1), 176-180. DOI: https://doi.org/10.1002/adma.201402471.
